# “Keep it simple, scholar”: an experimental analysis of few-parameter segmentation networks for retinal vessels in fundus imaging

**DOI:** 10.1007/s11548-021-02340-1

**Published:** 2021-04-30

**Authors:** Weilin Fu, Katharina Breininger, Roman Schaffert, Zhaoya Pan, Andreas Maier

**Affiliations:** 1 Pattern Recognition Lab, Friedrich-Alexander-Universität Erlangen-Nürnberg, Erlangen, Germany; 2 Erlangen Graduate School in Advanced Optical Technologies, Erlangen, Germany; 3 International Max Planck Research School for Physics of Light, Erlangen, Germany

**Keywords:** U-Net, Vessel segmentation, Fundus image, Computation cost

## Abstract

**Purpose:**

With the recent development of deep learning technologies, various neural networks have been proposed for fundus retinal vessel segmentation. Among them, the U-Net is regarded as one of the most successful architectures. In this work, we start with simplification of the U-Net, and explore the performance of few-parameter networks on this task.

**Methods:**

We firstly modify the model with popular functional blocks and additional resolution levels, then we switch to exploring the limits for compression of the network architecture. Experiments are designed to simplify the network structure, decrease the number of trainable parameters, and reduce the amount of training data. Performance evaluation is carried out on four public databases, namely DRIVE, STARE, HRF and CHASE_DB1. In addition, the generalization ability of the few-parameter networks are compared against the state-of-the-art segmentation network.

**Results:**

We demonstrate that the additive variants do not significantly improve the segmentation performance. The performance of the models are not severely harmed unless they are harshly degenerated: one level, or one filter in the input convolutional layer, or trained with one image. We also demonstrate that few-parameter networks have strong generalization ability.

**Conclusion:**

It is counter-intuitive that the U-Net produces reasonably good segmentation predictions until reaching the mentioned limits. Our work has two main contributions. On the one hand, the importance of different elements of the U-Net is evaluated, and the minimal U-Net which is capable of the task is presented. On the other hand, our work demonstrates that retinal vessel segmentation can be tackled by surprisingly simple configurations of U-Net reaching almost state-of-the-art performance. We also show that the simple configurations have better generalization ability than state-of-the-art models with high model complexity. These observations seem to be in contradiction to the current trend of continued increase in model complexity and capacity for the task under consideration.

**Supplementary Information:**

The online version contains supplementary material available at 10.1007/s11548-021-02340-1.

## Introduction

Retinal vessel segmentation from fundus images is an extensively studied field [14, 19, 40]. Analysis of the distribution, thickness and curvature of the retinal vessels assists the diagnosis, therapy planning, and treatment procedures of circulatory system-related eye diseases such as diabetic retinopathy (DR), glaucoma and age-related macular degeneration, which are the leading causes of blindness in the aging population [48]. Previous work on retinal vessel segmentation can be roughly divided into unsupervised and supervised categories, where supervised approaches often outperform the unsupervised ones. Unsupervised approaches do not require manual annotations, and are usually based on certain rules, such as template matching [4, 21, 45], vessel tracking [49, 54], region growing [35], multiscale analysis [3, 29, 51], and morphological processing [7]. Supervised approaches rely on ground truth annotations by expert ophthalmologists. In conventional machine learning-based methods, hand-crafted or learnt features are used as input for classifiers such as k-nearest neighbors (kNN) [46], support vector machine (SVM) [33], random forest (RF) [44], AdaBoost [8], Gaussian mixture model (GMM) [39], and the multilayer perceptron (MLP) [36]. With the recent advancements in deep learning-based technologies [27], convolutional neural networks (CNNs), which do not explicitly separate the feature extraction and the classification procedures, are employed in this field and have achieved great success [9, 25, 28]. Apart from models that are designed for high-performance, researchers have proposed to improve the interpretability of the constructed segmentation pipelines as well. For instance, the Frangi-Net [11], which is the CNN counterpart of the classical Frangi filter [6], has been proposed and combined with a preprocessing net [10] to reach the state-of-the-art performance.

Among the deep learning-based methods designed for biomedical image segmentation, U-Net [37] is one of the most successful models. Since published, U-Net and its variants have achieved remarkable performance in various applications and have been employed as the state-of-the-art method for segmentation tasks to compare with [23, 47, 52]. Isensee et al. [18] even draw an empirical conclusion that hyper-parameter tuning of the U-Net rather than new network architecture design is the key to high performance. Since the U-Net normally contains huge amounts of parameters, training and inference processes are resource-consuming. Compression of the network architecture has been tackled in previous work, such as the U-Net++ [55] by Zhou et al.. Additional convolutional layers are inserted in-between the skip connections to introduce self-similarity to the structure. This modification enables easy pruning in the testing phase, yet introduces parameters in the training phase. Besides, only one decisive structural factor, namely the number of levels, is considered.

This work is an extension of our previous publication [31], which focuses on degenerating the U-Net for retinal vessel segmentation on the DRIVE [41] database. The major differences comparing to [31] are as follows. Firstly, the U-Net variant with no skip connections is explored. Secondly, all experiments are conducted on three additional fundus databases besides the DRIVE [41], namely the STARE [15], the HRF [3], and the CHASE_DB1 [34]. Fourfold cross-validation is performed on these databases. Thirdly, parameter searching is conducted for training the default U-Net on the HRF database, which contains the largest number of fundus images, to explore how the hyperparameters affect the training process. Fourthly, a five-level U-Net is trained on the HRF database to explore how enlarging the model influences the performance. Lastly, the performance and generalization ability of our few-parameter nets are compared with that of the SSA-Net [32], which yields state-of-the-art performance on multiple fundus databases.

**Fig. 1:**
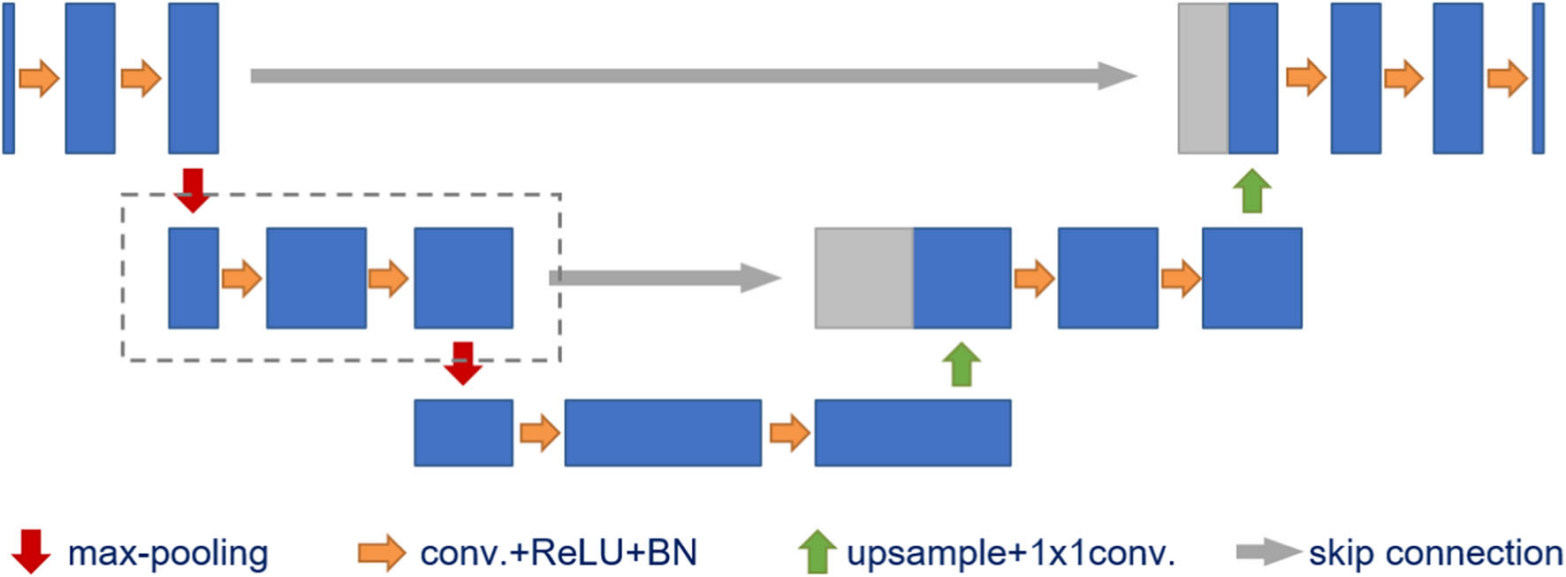
Default U-Net configuration. The dash box defines one U-Net block

**Fig. 2:**
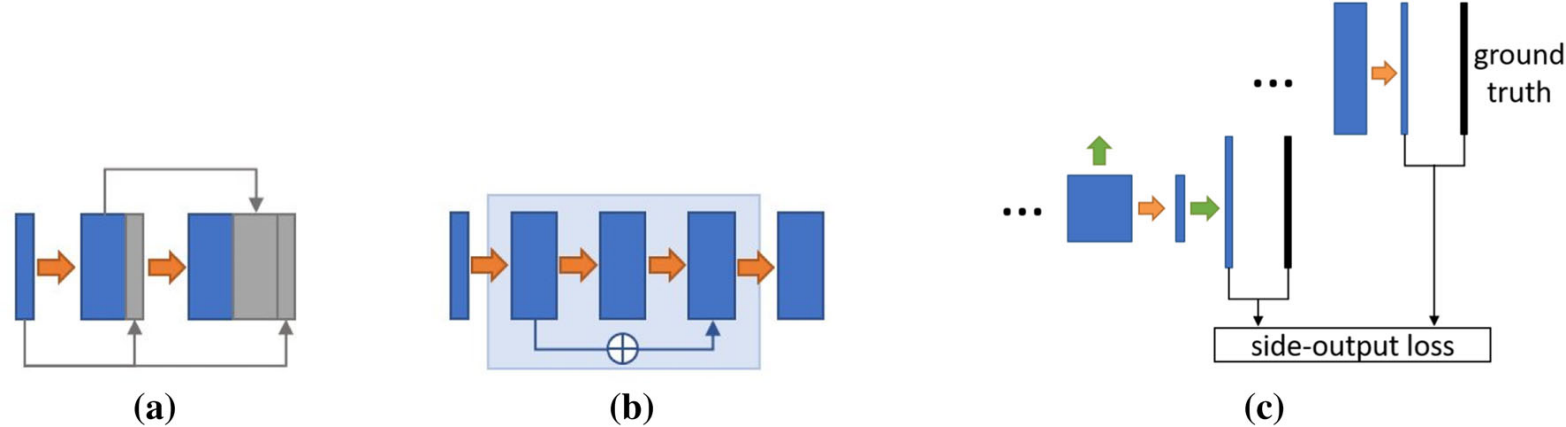
Illustration of the dense block (**a**), residual block (**b**), and the side-output block (**c**)

We start with a default U-Net and firstly seek to enhance its performance by introducing additional resolution scales and substituting the vanilla U-Net blocks with commonly used functional blocks, namely the dense block [16], the residual block [13], the dilated convolution block [50], and the side-output block [9]. Due to the observation of no remarkable performance boost, we propose the assumption that the default U-Net alone is capable or even over-qualified for the task of retinal vessel segmentation. Thereafter, we turn our focus onto simplification of the network architecture, aiming for a minimized model which yields reasonably good performance. Different components of the default U-Net are explored independently using the “control variates” strategy, where only one factor is changed while the others are fixed at one time. The number of U-Net levels, the number of convolutional layers in each U-Net block, and the number of filters in the convolution layers are step-wise decreased; the nonlinear activation layers and skip connections are removed; and the size of training set is reduced. Analysis of the performance evaluation metrics yields unexpected conclusion; only under substantially harsh conditions does the U-Net degenerate. With one down-/upsampling step, or one convolutional layer in each U-Net block, or two filters in the input layer, the segmentation performance remain satisfactory, producing AUC scores above 0.97. Comparison to the SSA-Net [32], which is state-of-the-art retinal vessel segmentation network model, also reveals that the few-parameter networks have strong generalization ability. The contribution of this work is two-sided. On the one hand, the importance of different configuration components of the U-Net model is quantitatively assessed, and a minimized well-performing model is obtained. On the other hand, this work provides an exemplary reminder that the research behavior of pursuing marginal performance gain at the cost of massive resource consumption could be unworthy.

## Materials and methods

### Default U-Net configuration

The default U-Net configuration in this work is illustrated in Fig. 1. Likewise the original U-Net [37], each U-Net block consists of two consecutive convolutional layers with $3\times 3$ filters. The number of filters doubles after each down-sampling, and halves after each up-sampling. Down-sampling is performed by the max-pooling operation. ReLU activation layers are employed to introduce nonlinearity into the model, and the concatenation operation is used as the skip connection to merge the localization and contextual information. In comparison to the original U-Net architecture, four major modifications are made. Firstly, our model is composed of three rather than five scale levels. Secondly, the number of filters in the first convolutional layer is set to 16 rather than 64. Thirdly, up-sampling is realized with an up-pooling layer followed by a $1\times 1$ convolutional layer rather than the transposed convolutional layer. Lastly, batch normalization [17] layers are applied after all but the last ReLU [31] layers to stabilize the training process. The overall architecture contains 108,976 parameters.

### Additive variants

Four structural additive modifications are applied on the vanilla U-Net architecture, namely the dense block [16], the residual block [13], the side-output block [9] (see Fig. 2), and the dilated convolution block [50]. These structural modifications are chosen due to their popularity in the U-Net-based medical image segmentation community [1, 5, 22, 23, 26, 30, 43, 53]. In the dense block, activation maps from all preceding layers are concatenated to all latter ones. Such connections create many additional channels and introduce a large amount of parameters. Due to computational resource limits, dense blocks replace the vanilla blocks only in the encoder path. In the residual block, two additional convolutional layers are inserted, where the activation maps from the first convolutional layer are added to those of the third layer. The residual blocks replace the vanilla U-Net blocks in the encoder, the bottleneck, as well as the decoder. The concatenation operations in dense blocks and the addition operations in residual blocks allow for better gradient backpropagation since preceding layers can receive more direct supervision from the loss function. In dilated convolution layers, the kernels are enlarged, creating holes in-between which are filled with zeros. No additional parameters are introduced, while the receptive field is enlarged. The dilated convolution block is employed in the bottleneck of the model. The side-output blocks are applied in the decoder path to provide step-wise deep supervision, where the output maps from the U-Net blocks are passed through a $1\times 1$ convolutional layer, upsampled to the shape of the network input, and compared with the ground truth using a mean square error (MSE) loss. Besides, a U-Net with five scale levels is trained on the biggest fundus database, namely the HRF [3] database to explore how enlarged architecture influences the network performance.

### Subtractive variants

The default U-Net in this study is configured as described in “Default U-Net configuration” section. Exploration of the limits of subtractive U-Net variants follows the “control variates” strategy, which means only one aspect of the model is changed from the default configuration at one time. Experiment series are designed as:

1. Nonlinear activation functions, i.e., the ReLU layers, are removed.
2. Skip connections between the encoder and the decoder are removed.
3. The number of convolutional layers in each U-Net block is reduced to one.
4. The number of filters in the first level is halved from sixteen down to one. Correspondingly, the number of filters in deep levels is proportionally decreased.
5. The number of levels decreases step-wise to one, until the network degenerates into a chain of convolutional layers.
6. The number of images for training the model is consecutively halved by a factor of two until only one image is used.

### Parameter searching

In order to investigate on the importance of parameter tuning for the network performance, a random hyperparameter searching [2] experiment is carried out for the default U-Net configuration on the HRF [3] database which contains the largest number of annotated fundus images. Nine different hyperparameters which control the model architecture and the training process are considered. The optimum parameter combination is selected from 29 experiment roll-outs, and utilized to retrain the default U-Net. The experimental details for parameter searching are elaborated in the supplementary material.

### Comparison to the state-of-the-art method

To compare the performance of our few-parameter networks with the state-of-the-art methods, we select the scale-space approximated network [32] (SSA-Net) which reaches the highest performance on various fundus databases as the target model. We firstly rerun the SSA-Net for five repetitive times to obtain the mean and standard deviation of the experiments rather than merely the optimum results as in [32]. Note that the SSA-Net is trained with the exactly same software and configuration as in [32]. Since the SSA-Net utilizes the backbone of ResNet34 [13] and contains more than 25 million trainable weights, it is natural to propose that the high performance of the model could be due to overfitting. Thereafter an experiment to investigate on the generalization ability of the network models is designed. Both our few-parameter networks and the SSA-Net are trained on the DRIVE database and transferred to the STARE [15] directly.

### Database description

#### DRIVE

The digital retinal images for vessel extraction (DRIVE) [41] database contains 40 8-bit RGB fundus images with a resolution of $565\times 584$ pixels. The database consists of 33 healthy cases and 7 cases with early signs of DR, and is evenly divided into one training and one testing set. In this work, a subset of four images is further separated from the training set for validation purpose. For all images, FOV masks and manually labeled annotations are provided. In the training process, each minibatch contains 50 image patches of size $168\times 168$, which are randomly sampled from the training images.

**Fig. 3:**
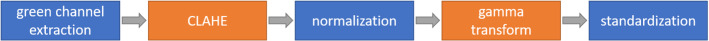
Preprocessing pipeline

#### STARE

The structured analysis of the retina (STARE) database [15] contains 20 8-bit RGB fundus photographs of size $605\times 700$ pixels. Half of the images are from healthy subjects, while the other half is corrupted with pathologies that affect the visibility of retinal vessels. Manually labeled vessel masks are available for all images. FOV masks are generated using a foreground / background separation technique named “GrabCut” [38]. Training and testing sets are not predefined. A fourfold cross-validation is performed, with five images for testing, eleven images for training and four images for validation in each experiment. During the training process, minibatches are constructed in the same way as for DRIVE.

**Fig. 4:**
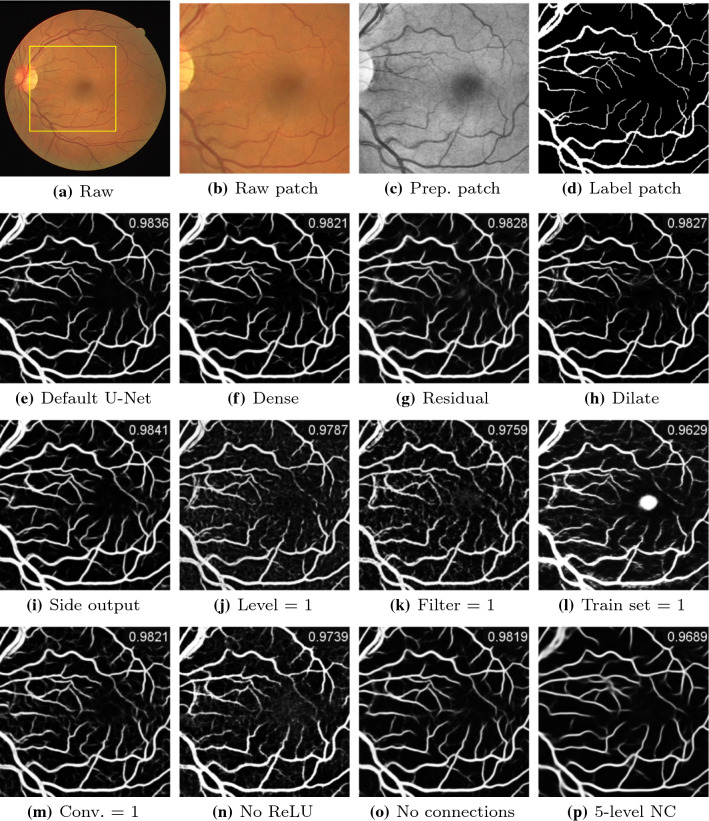
Probability predictions of U-Net variants with AUC scores presented on upper right corners. (**f–i**) are the additive variants of the U-Net. (**j–m**) denote U-Net with one level, U-Net with one filter in the initial convolutional layer, U-Net trained with one sample, and U-Net with one convolutional layer in each block. (**n–p**) correspond to U-Net without ReLU layers, three-level U-Net without skip connections, and five-level U-Net without skip connections

#### HRF

The high-resolution fundus (HRF) image database [3] consists of 45 8-bit RGB fundus photographs of size $2336\times 3504$ pixels. It contains 15 images from healthy patients, 15 from DR patients, and 15 from glaucomatous patients. For each image, a manual annotation and an FOV mask are provided. Training and testing sets are not predefined, and a fourfold cross-validation is performed for evaluation. In each experiment, 34 images are used for training, seven for validation, and eleven/twelve for testing. In the training process, each minibatch contains 15 patches of size $400\times 400$ pixels.

#### CHASE_DB1

The CHASE_DB1 [34] database contains 28 fundus images from both eyes of 14 pediatric subjects with a resolution of $999\times 960$ pixels. Ground truth vessel maps are provided, yet FOV masks are created using the GrabCut algorithm. For evaluation, a fourfold cross-validation is performed. The 28 images are divided into a training set of 17 images, a validation set of four images, and testing set containing seven images in each experiment. For training, a minibatch contains 40 patches of shape $200\times 200$ pixels.

### Preprocessing pipeline

Before fed into network models, raw fundus photographs are preprocessed using the pipeline illustrated in Fig. 3. Firstly, the green channels of the RGB images, which exhibit the best contrast between the retinal vessels and the background, are extracted. Secondly, the CLAHE [56] algorithm, with a window size of $8\times 8$ pixels and the max slope equals 3.0, is applied to equalize the local histogram in an adaptive manner and balance the illumination. The data range within the FOV masks is then normalized between 0.0 and 1.0, and a Gamma transform with $\gamma = 0.8$ is applied to further lift the contrast in dark small vessel regions. Finally, the data range within the FOV mask is standardized between $-1.0$ and 1.0 to generate input for the networks. Additionally for HRF and CHASE_DB1 databases, images are down-sampled with bilinear interpolation by a factor of 4 and 2, respectively, before fed into networks, and up-scaled after the network processing to restore their original shape.

The borders of FOV masks of all databases are inwardly eroded by four pixels to remove potential border effects and ensure meaningful comparison. In order to stress on the thin vessels during training, weight maps are generated and multiplied to the pixel-wise loss as in Eq. (1), where $d_{x_i}$ is the vessel diameter in the manual label map of the given pixel $x_i$:

1 $$\begin{aligned} W(x_i) = \left\{ \begin{array}{ll} 1.0, &{} {\text { if }}\; x_i \;{\text { in background,}}\\ {\max }(1.0, \frac{1.0}{0.18\cdot d_{x_i}}), &{} {\text { if }}\; x_i \;{\text { in foreground,}} \end{array} \right. \end{aligned}$$

### Experimental details

The objective function in this work is a weighted sum of two parts, namely the segmentation loss and the regularization loss, i.e.,

2 $$\begin{aligned} L = L_{{\text {seg}}} + L_{{\text {reg}}} = \frac{1}{N}\cdot \sum _{i=1}^{N}(L_{{\text {focal}}}(x_i)\cdot W(x_i)) + \lambda \cdot L_{{\ell }_2}, \end{aligned}$$

where $L_{\mathrm{focal}}(x_i)$ is the focal loss [24] for a given pixel $x_i$, *N* is the overall number of pixels, and $L_{{\ell }_2}$ is the regularizer loss representing the $\ell _2$ norm of all network weights. For the focal loss, the focusing factor $\gamma$ is set to 2.0 to differentiate between easy and hard cases, and a class-balancing factor $\alpha$ is set to 0.9 to emphasize on the foreground pixels. The $\ell _2$ loss is combined with the segmentation loss with a factor $\lambda =0.2$ to prevent over-fitting. The Adam optimizer [20] with $\beta _1 = 0.9, \beta _2=0.999$ is used for the training process. The learning rate decays by 10% after each 10,000 iterations. Different initial learning rates are tailored for different models to achieve smooth loss curves; the more weights in the model, the smaller the learning rate. Networks are trained until convergence is observed in the validation loss curve. Data augmentation techniques are utilized for better generalization, including rotation within 20 degrees, shearing within 30% of the linear patch size, zooming between 50% and 150% of the linear patch size, additive Gaussian noise and uniform intensity shifting within the range of 8% of the image intensities.

**Table 1:** Performance w.r.t. structural variants. Additive variants: Ures, Uden, Udil, Uside denote the U-Net with the residual blocks, U-Net with the dense blocks, U-Net with the dilate convolution block, U-Net with the side-output block; subtractive variants: U-lin, U-1C, U-ns represent U-Net without ReLU layers and U-Net with one convolutional layer per level, and U-Net without skip connections, respectively. U-par, U-5lv, and SSA represent default U-Net with parameter searching, five-level U-Net and the SSA-Net, respectively

Model	Parameter	AUC	Specificity	Sensitivity	F1 score	Accuracy
DRIVE						
U	108,976	0.9756 ± 0.0010	0.9758 ± 0.0016	0.7941 ± 0.0073	0.8101 ± 0.0032	0.9518 ± 0.0009
Ures	154,768	0.9765 ± 0.0009	0.9758 ± 0.0009	0.7994 ± 0.0053	0.8133 ± 0.0034	0.9525 ± 0.0008
Uden	2,501,067	0.9754 ± 0.0009	0.9742 ± 0.0017	0.8029 ± 0.0063	0.8110 ± 0.0042	0.9515 ± 0.0012
Udil	108,976	0.9741 ± 0.0013	0.9753 ± 0.0030	0.7944 ± 0.0151	0.8089 ± 0.0047	0.9513 ± 0.0014
Uside	109,072	0.9752 ± 0.0008	0.9757 ± 0.0013	0.7938 ± 0.0073	0.8097 ± 0.0033	0.9517 ± 0.0008
U-lin	108,976	0.9643 ± 0.0016	0.9693 ± 0.0024	0.7874 ± 0.0091	0.7885 ± 0.0035	0.9453 ± 0.0012
U-ns	97,456	0.9752 ± 0.0009	0.9745 ± 0.0015	0.7966 ± 0.0068	0.8082 ± 0.0036	0.9510 ± 0.0010
U-1C	49,072	0.9732 ± 0.0009	0.9742 ± 0.0010	0.7918 ± 0.0055	0.8043 ± 0.0028	0.9501 ± 0.0007
SSA	25,879,328	0.9810 ± 0.0004	0.9774 ± 0.0009	0.8205 ± 0.0051	0.8306 ± 0.0009	0.9567 ± 0.0002
STARE						
U	108,976	0.9835 ± 0.0012	0.9813 ± 0.0017	0.7997 ± 0.0114	0.8115 ± 0.0059	0.9621 ± 0.0012
Ures	154,768	0.9836 ± 0.0012	0.9812 ± 0.0015	0.8024 ± 0.0096	0.8132 ± 0.0051	0.9624 ± 0.0011
Uden	2,501,067	0.9796 ± 0.0019	0.9822 ± 0.0013	0.7885 ± 0.0088	0.8075 ± 0.0046	0.9618 ± 0.0009
Udil	108,976	0.9838 ± 0.0023	0.9799 ± 0.0028	0.8092 ± 0.0181	0.8129 ± 0.0122	0.9620 ± 0.0023
Uside	109,072	0.9829 ± 0.0014	0.9816 ± 0.0017	0.7978 ± 0.0100	0.8110 ± 0.0050	0.9621 ± 0.0011
U-lin	108,976	0.9734 ± 0.0044	0.9788 ± 0.0029	0.7556 ± 0.0257	0.7723 ± 0.0149	0.9554 ± 0.0025
U-ns	97,456	0.9853 ± 0.0036	0.9807 ± 0.0057	0.8064 ± 0.0229	0.8149 ± 0.0187	0.9623 ± 0.0048
U-1C	49,072	0.9825 ± 0.0011	0.9815 ± 0.0015	0.7808 ± 0.0099	0.7997 ± 0.0052	0.9602 ± 0.0011
HRF						
U	108,976	0.9810 ± 0.0010	0.9761 ± 0.0010	0.7921 ± 0.0073	0.7754 ± 0.0041	0.9590 ± 0.0008
Ures	154,768	0.9820 ± 0.0008	0.9764 ± 0.0009	0.7953 ± 0.0058	0.7785 ± 0.0031	0.9595 ± 0.0007
Uden	2,501,067	0.9821 ± 0.0006	0.9768 ± 0.0007	0.7949 ± 0.0060	0.7799 ± 0.0029	0.9599 ± 0.0006
Udil	108,976	0.9816 ± 0.0006	0.9765 ± 0.0013	0.7951 ± 0.0084	0.7788 ± 0.0034	0.9596 ± 0.0008
Uside	109,072	0.9822 ± 0.0007	0.9762 ± 0.0008	0.7980 ± 0.0061	0.7793 ± 0.0040	0.9595 ± 0.0008
U-lin	108,976	0.9641 ± 0.0069	0.9712 ± 0.0035	0.7599 ± 0.0216	0.7388 ± 0.0117	0.9519 ± 0.0025
U-ns	97,456	0.9815 ± 0.0007	0.9764 ± 0.0010	0.7926 ± 0.0081	0.7771 ± 0.0038	0.9593 ± 0.0008
U-1C	49,072	0.9779 ± 0.0023	0.9756 ± 0.0022	0.7804 ± 0.0136	0.7668 ± 0.0095	0.9575 ± 0.0019
U-par	108,976	0.9825 ± 0.0007	0.9767 ± 0.0010	0.7976 ± 0.0065	0.7809 ± 0.0033	0.9600 ± 0.0007
U-5lv	1,852,336	0.9831 ± 0.0006	0.9766 ± 0.0006	0.8004 ± 0.0050	0.7823 ± 0.0024	0.9602 ± 0.0005
CHASE_DB1						
U	108,976	0.9806 ± 0.0010	0.9731 ± 0.0013	0.8225 ± 0.0073	0.7964 ± 0.0045	0.9575 ± 0.0011
Ures	154,768	0.9811 ± 0.0011	0.9737 ± 0.0015	0.8231 ± 0.0088	0.7987 ± 0.0049	0.9581 ± 0.0011
Uden	2,501,067	0.9799 ± 0.0010	0.9734 ± 0.0013	0.8180 ± 0.0068	0.7951 ± 0.0041	0.9574 ± 0.0010
Udil	108,976	0.9783 ± 0.0020	0.9734 ± 0.0020	0.8120 ± 0.0129	0.7921 ± 0.0066	0.9569 ± 0.0015
Uside	109,072	0.9806 ± 0.0010	0.9737 ± 0.0013	0.8174 ± 0.0084	0.7955 ± 0.0056	0.9576 ± 0.0012
U-lin	108,976	0.9619 ± 0.0047	0.9639 ± 0.0041	0.7910 ± 0.0180	0.7475 ± 0.0098	0.9462 ± 0.0029
U-ns	97,456	0.9793 ± 0.0009	0.9728 ± 0.0011	0.8145 ± 0.0061	0.7907 ± 0.0032	0.9564 ± 0.0008
U-1C	49,072	0.9773 ± 0.0012	0.9713 ± 0.0013	0.8096 ± 0.0070	0.7826 ± 0.0041	0.9546 ± 0.0010

**Table 2:** U-Net performance w.r.t. different numbers of initial filters

#	Parameter	AUC	Specificity	Sensitivity	F1 score	Accuracy
DRIVE						
8	27,352	0.9754 ± 0.0008	0.9754 ± 0.0012	0.7940 ± 0.0055	0.8089 ± 0.0036	0.9514 ± 0.0010
4	6892	0.9748 ± 0.0007	0.9746 ± 0.0012	0.7962 ± 0.0056	0.8080 ± 0.0025	0.9510 ± 0.0007
2	1750	0.9719 ± 0.0008	0.9728 ± 0.0009	0.7889 ± 0.0047	0.7986 ± 0.0021	0.9485 ± 0.0005
1	451	0.9637 ± 0.0014	0.9678 ± 0.0030	0.7776 ± 0.0110	0.7785 ± 0.0052	0.9427 ± 0.0018
STARE						
8	27,352	0.9831 ± 0.0010	0.9812 ± 0.0013	0.7900 ± 0.0101	0.8052 ± 0.0053	0.9610 ± 0.0010
4	6892	0.9824 ± 0.0010	0.9811 ± 0.0013	0.7806 ± 0.0082	0.7988 ± 0.0047	0.9599 ± 0.0009
2	1750	0.9787 ± 0.0018	0.9794 ± 0.0017	0.7605 ± 0.0118	0.7799 ± 0.0078	0.9562 ± 0.0015
1	451	0.9752 ± 0.0016	0.9772 ± 0.0018	0.7405 ± 0.0119	0.7595 ± 0.0081	0.9522 ± 0.0017
HRF						
8	27,352	0.9811 ± 0.0008	0.9763 ± 0.0009	0.7913 ± 0.0056	0.7760 ± 0.0038	0.9591 ± 0.0008
4	6892	0.9801 ± 0.0009	0.9762 ± 0.0008	0.7897 ± 0.0070	0.7744 ± 0.0038	0.9589 ± 0.0007
2	1750	0.9762 ± 0.0010	0.9752 ± 0.0011	0.7771 ± 0.0074	0.7633 ± 0.0037	0.9568 ± 0.0008
1	451	0.9679 ± 0.0014	0.9735 ± 0.0016	0.7520 ± 0.0111	0.7424 ± 0.0054	0.9531 ± 0.0012
CHASE_DB1						
8	27,352	0.9798 ± 0.0010	0.9733 ± 0.0014	0.8169 ± 0.0085	0.7938 ± 0.0039	0.9571 ± 0.0009
4	6892	0.9788 ± 0.0009	0.9723 ± 0.0012	0.8133 ± 0.0069	0.7884 ± 0.0038	0.9559 ± 0.0009
2	1750	0.9734 ± 0.0015	0.9693 ± 0.0016	0.7966 ± 0.0090	0.7686 ± 0.0048	0.9515 ± 0.0011
1	451	0.9615 ± 0.0023	0.9622 ± 0.0042	0.7633 ± 0.0147	0.7269 ± 0.0090	0.9480 ± 0.0032

**Table 3:** U-Net performance w.r.t. different numbers of levels

#	Parameter	AUC	Specificity	Sensitivity	F1 score	Accuracy
DRIVE						
2	23,984	0.9735 ± 0.0006	0.9733 ± 0.0017	0.7970 ± 0.0072	0.8050 ± 0.0027	0.9500 ± 0.0009
1	7344	0.9649 ± 0.0007	0.9652 ± 0.0015	0.7970 ± 0.0060	0.7832 ± 0.0026	0.9429 ± 0.0008
STARE						
2	23,984	0.9813 ± 0.0011	0.9820 ± 0.0013	0.7645 ± 0.0090	0.7912 ± 0.0046	0.9590 ± 0.0009
1	7344	0.9702 ± 0.0012	0.9759 ± 0.0011	0.7235 ± 0.0090	0.7413 ± 0.0062	0.9494 ± 0.0012
HRF						
2	23,984	0.9794 ± 0.0008	0.9760 ± 0.0011	0.7891 ± 0.0070	0.7736 ± 0.0034	0.9587 ± 0.0008
1	7344	0.9690 ± 0.0029	0.9741 ± 0.0019	0.7520 ± 0.0139	0.7448 ± 0.0086	0.9537 ± 0.0018
CHASE_DB1						
2	23,984	0.9771 ± 0.0011	0.9731 ± 0.0013	0.8021 ± 0.0075	0.7844 ± 0.0036	0.9555 ± 0.0008
1	7344	0.9679 ± 0.0023	0.9685 ± 0.0017	0.7746 ± 0.0087	0.7533 ± 0.0058	0.9487 ± 0.0014

**Table 4:** U-Net performance w.r.t. various number of training images

#	AUC	Specificity	Sensitivity	F1 score	Accuracy
DRIVE					
8	0.9734 ± 0.0013	0.9732 ± 0.0025	0.7961 ± 0.0120	0.8043 ± 0.0050	0.9498 ± 0.0014
4	0.9686 ± 0.0019	0.9700 ± 0.0041	0.7926 ± 0.0161	0.7935 ± 0.0065	0.9465 ± 0.0021
2	0.9654 ± 0.0032	0.9657 ± 0.0080	0.7919 ± 0.0220	0.7818 ± 0.0123	0.9427 ± 0.0049
1	0.9564 ± 0.0068	0.9672 ± 0.0058	0.7508 ± 0.0274	0.7602 ± 0.0192	0.9387 ± 0.0054
STARE					
5	0.9753 ± 0.0042	0.9789 ± 0.0035	0.7780 ± 0.0253	0.7890 ± 0.0149	0.9576 ± 0.0028
2	0.9614 ± 0.0030	0.9709 ± 0.0023	0.7413 ± 0.0110	0.7400 ± 0.0073	0.9463 ± 0.0018
1	0.9511 ± 0.0045	0.9709 ± 0.0026	0.7127 ± 0.0138	0.7197 ± 0.0108	0.9435 ± 0.0023
HRF					
14	0.9817 ± 0.0007	0.9764 ± 0.0011	0.7934 ± 0.0071	0.7774 ± 0.0038	0.9593 ± 0.0008
7	0.9805 ± 0.0010	0.9755 ± 0.0015	0.7913 ± 0.0096	0.7730 ± 0.0051	0.9584 ± 0.0011
3	0.9779 ± 0.0017	0.9750 ± 0.0021	0.7804 ± 0.0149	0.7644 ± 0.0083	0.9569 ± 0.0018
1	0.9727 ± 0.0026	0.9724 ± 0.0026	0.7626 ± 0.0200	0.7441 ± 0.0127	0.9529 ± 0.0026
CHASE_DB1					
8	0.9771 ± 0.0015	0.9718 ± 0.0022	0.8081 ± 0.0109	0.7833 ± 0.0060	0.9549 ± 0.0015
4	0.9728 ± 0.0020	0.9703 ± 0.0030	0.7953 ± 0.0123	0.7707 ± 0.0091	0.9522 ± 0.0023
2	0.9684 ± 0.0037	0.9693 ± 0.0028	0.7847 ± 0.0148	0.7609 ± 0.0115	0.9502 ± 0.0027
1	0.9590 ± 0.0059	0.9659 ± 0.0045	0.7631 ± 0.0170	0.7366 ± 0.0169	0.9449 ± 0.0044

Experiments with each different configuration are repeated for five times to make sure that the conclusion is not dominated by certain specific initialization settings, and to evaluate the stability of the model. The models are trained on an NVIDIA GPU cluster. Projects are implemented in Python 3.6.8., using the framework TensorFlow 1.13.1.

## Results

Commonly used performance evaluation metrics for semantic medical image segmentation, namely specificity, sensitivity, F1 score, accuracy and the AUC score [42], are employed in this work. Binarization of the prediction maps from a model is conducted by selecting a threshold which maximizes the average F1 score of the validation sets. The AUC score, which is threshold-independent, is chosen as the major performance indicator. The mean and standard deviation of the metric values on each testing image over the five experiment roll-outs are firstly computed individually. The average of these mean and standard deviation values over all the testing images are reported in Tables 1, 2, 3 and 4. The evaluation results to compare the generalization ability of our few-parameter networks with the SSA-Net are presented in Table 5. The significance analysis of predictions from different U-Net variants is presented in the supplementary material. The predicted probability maps from different network variants for one testing image in DRIVE are shown in Fig. 4a–o.

Performance evaluation of structural U-Net variants are presented in Table 1. For additive variants, we observe that comparing to the vanilla U-Net, the changes in AUC scores stay in reach of the standard deviations. This implies that the introduced functional blocks or the additional levels fail to incur the expected performance enhancement. As for the subtractive variants, the performance of U-Net with one convolutional layer in each block drops marginally and remains satisfactory. Removing skip connections barely harms the network performance; while eliminating the ReLU layer causes 0.01 decrease in the AUC scores. In Table 2, the evaluation metrics of the U-Nets with decreased number of filters in the initial convolutional layer are reported. A uniform performance decay is observed as the network shrinks. However, it is remarkable that the performance remains reasonable with AUC scores above 0.96 for all databases even for the model with a total of 451 parameters and with only one filter in the first convolutional layer. U-Nets with reduced number of levels are evaluated in Table 3. We notice that compared to the default three-level U-Net, the segmentation capability of the two-level U-Net is basically retained; and that even if the model degenerates into a chain of convolutional layers, the predictions remain plausible, reaching AUC scores above 0.96 for all databases. Experiment series of training the default U-Net with decreased amount of data in Table 4 show the generalization ability of the model. In accordance with expectation, a monotonous performance decline concurs with a decreasing number of samples in the training set. However, it is unexpected that the U-Nets trained with only two images achieve AUC scores above 0.96 in all databases.

## Discussion and conclusion

In this work, we firstly attempt to improve the capability of U-Net on the retinal vessel segmentation task by introducing functional blocks or additional scale levels to the model. Although the modified models accommodate more parameters, their performance does not improve considerably. To investigate on the impact of hyperparameters on the network performance, a parameter searching experiment is carried out for the default U-Net on the HRF database. However, the optimum set of parameters also fails to introduce significant improvement. Thereafter, we turn our research direction into exploring the minimum configurations of the U-Net by removing or reducing certain characteristics from a default U-Net configuration. It is proved that ReLU layers have larger impact on the model functionality than the amount of parameters. Linear U-Nets with no ReLU activation levels arrive at the lowest segmentation performance among all structural variants on all four databases. In the DRIVE database, the default U-Net achieves an AUC score of 0.9756, the U-Net with two filters in the input layer achieves an AUC score of 0.9719, while U-Net without ReLU layers yields an AUC score of 0.9643, as presented in Tables 1, 2. One interesting observation is that when skip connections are absent, the high performance is maintained. A possible explanation is that the detail loss due to resampling is limited in three-level models and that the missing details can still be successfully encoded in the bottleneck. In other words, for this specific task, skip connections are not necessary when the network is shallow. The assumption is confirmed by evaluating the segmentation performance on a five-level U-Net without skip connections. Comparing the prediction of the five-level linear U-Net in Fig. 4p and that of the three-level linear U-Net in Fig. 4o, we observe that qualitatively not only are thin vessels neglected, but adjacent big vessels get blended as well; and that quantitatively the AUC score drastically drops from 0.9819 to 0.9689 as exhibited on the upper right corners of corresponding image tiles.

The segmentation performance of U-Net-based few-parameter networks are compared with the state-of-the-art retinal vessel segmentation model SSA-Net. Although their model performance is significantly better than ours, the differences are on the third digit. Besides, the generalization ability is another issue. When trained on the DRIVE database and directly transferred to the STARE database, our few parameter models exhibit much stronger generalization ability than the SSA-Net. The AUC scores yielded from our models are all above 0.96, while that from the SSA-Net is around 0.94 as presented in Table 5. The poor generalization ability could be explained by overfitting since the SSA-Net contains more than 25 million trainable parameters which is over 250 times more than that of our default U-Net.

**Table 5:** The AUC scores of transferring each model that is trained on the DRIVE database directly onto the STARE database. Few-parameter networks include the three-level U-Net with different numbers of filters in the first convolutional layer, and U-Net with few levels

AUC	Default U	SSA-Net	8 filter	4 filter
	0.9760 ± 0.0041	0.9405 ± 0.0090	0.97426 ± 0.0044	0.9751 ± 0.0034
	2 filter	1 filter	2 level	1 level
AUC	0.9706 ± 0.0047	0.9667 ± 0.0031	0.9710 ± 0.0029	0.9648 ± 0.0026

The observation that U-Net produces pleasing segmentation predictions even under extreme configuration conditions is unanticipated and intriguing. Small networks save both memory and computational resource, and allow for agile usage on mobile devices. Given the fundamental network architecture, the performance gain caused by increasing the amount of parameters or training data becomes marginal once the corresponding conditions, namely the minimal number of levels, number of filters, and number of convolutional layer in each block, are sufficiently satisfied. On the one hand, this observation could be explained by the simplicity of the task and the similarity among fundus photographs; on the other hand, it raises the question whether trading immense resource cost with minor performance increase is worthwhile. As future work, the same “control variates” methodology could be applied on alternative tasks for compression. Smart rather than bulky design should be the preferred research direction.

## Supplementary Information

Below is the link to the electronic supplementary material.

Supplementary material 1 (pdf 74 KB)

## Data Availability

All databases utilized in this publication are publicly available, namely DRIVE [15], STARE [41], HRF [3], and CHASE_DB1 [34].

## Declarations

### Code availability

Not available.
