# Supplementary material for: “Keep it simple, scholar”: an experimental analysis of few-parameter segmentation networks for retinal vessels in fundus imaging
Source: Int J Comput Assist Radiol Surg. 2021 Apr 30;16(6):967–78. doi: 10.1007/s11548-021-02340-1 (PMC8166700; doi:10.1007/s11548-021-02340-1)
Supplement: Supplementary file 1 — Supplementary material 1 (pdf 74 KB) [file 11548_2021_2340_MOESM1_ESM.pdf]

Supplementary: “Keep it Simple, Scholar”: An Experimental Analysis of Few-Parameter Segmentation Networks for Retinal Vessels in Fundus Imaging

Weilin Fu, Katharina Breininger, Roman Schaffert, Zhaoya Pan, and Andreas Maier

I. Parameter Searching

The parameter searching experiment is carried out on the HRF [1] database, since it contains 45 images and is the largest public annotated fundus database. Nine hyperparameters are taken into consideration. The size of training image patches are selected from the pool of $304^2, 400^2, 496^2, 592^2, 704^2$. The size of the filter in each convolution operation is chosen from $3^2, 5^2, 7^2$. The size of the training batch ranges from 5 to 30. The optimizer is selected from the Adam optimizer [2], the SGD with momentum optimizer and the gradient descent optimizer. The learning rate is chosen from the the pool of $10^{-2}, 10^{-3}, 10^{-4}, 10^{-5}, 10^{-6}$. After 5000, 10000, 15000 or 20000 steps, the learning rate decays with a decaying rate ranging from 0.8 to 0.95. If the SGD with momentum optimizer is chosen, the momentum is a value that ranges between 0.6 and 0.9. The regularizer for the weights in the models ranges between 0.01 and 0.25. The training process goes on for 30000 iterations. The convergence speeds of the loss curves and absolute values of the F1 score curves of the validation sets are used as the indices to determine the quality of the training process, and the validation images are binarized with the threshold of 0.5. 29 experiment roll-outs with randomly selected hyperparameter combinations are conducted. The hyperparameters together with our observations are presented in Table I. The selected configuration is highlighted in red.

TABLE I
THE RANDOMLY SELECTED PARAMETER COMBINATIONS FOR THE 29 EXPERIMENT ROLL-OUTS.

	exp01	exp02	exp03	exp04	exp05	exp06	exp07	exp08	exp09	exp10
patch size	[704, 704]	[304, 304]	[496, 496]	[304, 304]	[496, 496]	[704, 704]	[592, 592]	[592, 592]	[496, 496]	[704, 704]
filter size	[7, 7]	[5, 5]	[3, 3]	[7, 7]	[3, 3]	[5, 5]	[3, 3]	[5, 5]	[7, 7]	[3, 3]
batch size	11	26	23	29	21	28	15	5	13	15
optimizer	momen	momen	gradi	gradi	momen	gradi	gradi	gradi	momen	momen
learning rate	0.001	1e-05	0.0001	0.001	1e-06	0.0001	0.001	0.001	0.001	0.001
momentum	0.7583	0.7267	-	-	0.6893	-	-	-	0.8975	0.7646
decay rate	0.9055	0.8809	0.8422	0.8516	0.8443	0.8908	0.9382	0.8159	0.9200	0.8361
decay step	5000	20000	5000	5000	20000	15000	10000	5000	10000	20000
regularizer	0.1846	0.2056	0.1961	0.1034	0.1726	0.0716	0.0146	0.1276	0.2122	0.0200
convergence	slow	super slow	very slow	super slow	super slow		slow	very slow	fast	alright
F1 after 30k	0.75	0.13	0.18	0.14	0.15		0.66	0.35	0.78	0.58

	exp11	exp12	exp13	exp14	exp15	exp16	exp17	exp18	exp19	exp20
patch size	[304, 304]	[496, 496]	[400, 400]	[496, 496]	[592, 592]	[704, 704]	[496, 496]	[704, 704]	[400, 400]	[304, 304]
filter size	[5, 5]	[7, 7]	[7, 7]	[3, 3]	[7, 7]	[5, 5]	[3, 3]	[7, 7]	[5, 5]	[3, 3]
batch size	13	11	19	26	27	10	7	20	20	11
optimizer	gradi	Adam	Adam	momen	gradi	gradi	momen	gradi	Adam	gradi
learning rate	0.0001	0.001	0.01	0.01	0.01	0.0001	0.001	0.001	0.01	0.001
momentum	0.6967	0.7432	0.8643	0.6022	0.7000	-	0.7106	-	-	-
decay rate	-	-	-	0.9318	-	0.8148	0.8269	0.9486	0.8853	0.802
decay step	5000	10000	15000	20000	5000	15000	10000	5000	5000	15000
regularizer	0.0751	0.0436	0.0299	0.1310	0.0935	0.2046	0.1166	0.0620	0.1843	0.2412
convergence	super slow	very fast	very fast	fast	fast	super slow	alright		very fast	slow
F1 after 30k	0.11	0.74	0.4-0.6	0.83	0.74	0.133	0.617		0.6	0.43

	exp21	exp22	exp23	exp24	exp25	exp26	exp27	exp28	exp29
patch size	[592, 592]	[496, 496]	[704, 704]	[304, 304]	[704, 704]	[704, 704]	[496, 496]	[400, 400]	[592, 592]
filter size	[5, 5]	[3, 3]	[3, 3]	[7, 7]	[3, 3]	[3, 3]	[3, 3]	[7, 7]	[3, 3]
batch size	6	24	15	19	13	28	23	17	27
optimizer	momen	momen	Adam	gradi	gradi	Adam	momen	Adam	momen
learning rate	0.001	0.001	0.0001	0.01	0.0001	0.0001	0.0001	0.0001	0.0001
momentum	0.8786	0.7531	-	-	-	-	0.6419	-	0.7288
decay rate	0.8135	0.8674	0.9209	0.8348	0.9196	0.8708	0.9174	0.9092	0.811
decay step	10000	10000	5000	15000	20000	5000	10000	5000	10000
regularizer	0.1343	0.1503	0.0843	0.2132	0.0301	0.8708	0.056	0.2237	0.0167
convergence	fast	ok	fast	very fast	very slow	fast	slow	fast	very slow
F1 after 30k	0.74	0.68	0.77	0.67	0.1	0.75	0.2	0.79	0.2

II. SIGNIFICANCE TEST

The average AUC scores over five repetitive runs for each image in the HRF database on different U-Net variants are presented in Table II. The AUC scores of the predictions from each network configuration, which correspond to different columns of the table, are regarded as samples that are drawn from a distribution. Student t-tests [3] are carried out to judge whether the predictions from the U-Net variants are significantly different from those of the default U-Net configuration. Double tail t-tests assuming unequal variances with the alpha values equal 0.05 are performed. The null hypothesis for the tests is that the means of the distribution from the two distributions are the same. The calculated t-value is compared with the t-table value. When the computed t-value has a larger absolute value than the t-table value at the alpha level, we reject the hypothesis that the two distributions have the same mean value. In other words, the mean of the two distributions are significantly different. From Table III, it is observed that the U-Net with structural modifications, fine-tuned hyperparameters or additional scale levels are not significantly better than the default U-Net model as for segmentation performance.

TABLE II
THE AVERAGE AUC SCORES OVER FIVE REPETITIVE RUNS FOR EACH IMAGE ON DIFFERENT U-NET VARIANTS.

test_file	default U	residual	dense	dilate	side	conv1	linear	no skip	par	5-level
11_g	0.9831	0.9835	0.9824	0.9817	0.9831	0.9798	0.9696	0.9821	0.9829	0.9832
15_d	0.9782	0.9780	0.9780	0.9776	0.9782	0.9727	0.9574	0.9780	0.9775	0.9789
12_h	0.9873	0.9870	0.9874	0.9873	0.9872	0.9855	0.9797	0.9863	0.9873	0.9869
13_g	0.9786	0.9789	0.9781	0.9784	0.9789	0.9741	0.9619	0.9780	0.9791	0.9792
14_g	0.9769	0.9770	0.9764	0.9763	0.9769	0.9713	0.9585	0.9760	0.9774	0.9771
7_h	0.9894	0.9894	0.9887	0.9888	0.9892	0.9869	0.9789	0.9882	0.9891	0.9893
14_h	0.9878	0.9880	0.9880	0.9878	0.9877	0.9855	0.9761	0.9868	0.9880	0.9875
14_d	0.9756	0.9755	0.9747	0.9730	0.9754	0.9707	0.9558	0.9745	0.9755	0.9763
7_g	0.9844	0.9844	0.9834	0.9823	0.9846	0.9811	0.9712	0.9833	0.9841	0.9844
15_g	0.9790	0.9793	0.9782	0.9773	0.9793	0.9750	0.9622	0.9783	0.9789	0.9792
5_h	0.9890	0.9888	0.9882	0.9867	0.9887	0.9861	0.9785	0.9876	0.9886	0.9887
6_g	0.9851	0.9846	0.9852	0.9852	0.9846	0.9810	0.9687	0.9846	0.9857	0.9863
1_h	0.9810	0.9813	0.9816	0.9812	0.9816	0.9791	0.9603	0.9797	0.9818	0.9831
3_h	0.9805	0.9807	0.9812	0.9812	0.9806	0.9772	0.9556	0.9795	0.9812	0.9828
4_d	0.9800	0.9795	0.9807	0.9811	0.9805	0.9740	0.9597	0.9791	0.9807	0.9829
2_g	0.9826	0.9828	0.9828	0.9824	0.9827	0.9796	0.9673	0.9820	0.9830	0.9839
10_d	0.9788	0.9791	0.9794	0.9786	0.9791	0.9758	0.9603	0.9783	0.9794	0.9803
11_d	0.9822	0.9822	0.9825	0.9818	0.9823	0.9795	0.9643	0.9813	0.9825	0.9835
7_d	0.9809	0.9813	0.9812	0.9813	0.9811	0.9778	0.9640	0.9802	0.9815	0.9828
2_h	0.9866	0.9870	0.9870	0.9883	0.9871	0.9864	0.9782	0.9861	0.9873	0.9884
1_g	0.9783	0.9788	0.9788	0.9787	0.9785	0.9760	0.9625	0.9783	0.9788	0.9798
1_d	0.9712	0.9721	0.9723	0.9742	0.9716	0.9685	0.9531	0.9704	0.9729	0.9752
10_g	0.9829	0.9829	0.9834	0.9826	0.9836	0.9804	0.9665	0.9832	0.9837	0.9840
5_g	0.9854	0.9861	0.9866	0.9857	0.9868	0.9776	0.9686	0.9863	0.9867	0.9874
9_d	0.9680	0.9713	0.9718	0.9702	0.9724	0.9643	0.9325	0.9708	0.9733	0.9730
13_d	0.9800	0.9816	0.9822	0.9815	0.9824	0.9790	0.9604	0.9819	0.9827	0.9830
12_g	0.9808	0.9808	0.9811	0.9805	0.9815	0.9771	0.9641	0.9806	0.9814	0.9818
6_d	0.9585	0.9584	0.9618	0.9583	0.9621	0.9504	0.9252	0.9611	0.9636	0.9643
13_h	0.9858	0.9867	0.9878	0.9876	0.9868	0.9802	0.9712	0.9869	0.9876	0.9879
12_d	0.9736	0.9753	0.9764	0.9753	0.9763	0.9700	0.9500	0.9759	0.9770	0.9776
10_h	0.9834	0.9828	0.9839	0.9828	0.9842	0.9771	0.9603	0.9838	0.9843	0.9848
8_h	0.9873	0.9872	0.9879	0.9875	0.9879	0.9820	0.9710	0.9872	0.9879	0.9886
4_h	0.9861	0.9867	0.9885	0.9882	0.9873	0.9786	0.9650	0.9875	0.9880	0.9888
9_h	0.9827	0.9852	0.9850	0.9849	0.9853	0.9804	0.9655	0.9851	0.9853	0.9863
5_d	0.9815	0.9844	0.9844	0.9840	0.9845	0.9809	0.9696	0.9843	0.9845	0.9851
15_h	0.9885	0.9901	0.9895	0.9896	0.9899	0.9886	0.9780	0.9890	0.9897	0.9898
11_h	0.9868	0.9879	0.9873	0.9880	0.9877	0.9860	0.9770	0.9873	0.9878	0.9879
9_g	0.9796	0.9822	0.9823	0.9816	0.9823	0.9784	0.9657	0.9813	0.9823	0.9828
8_g	0.9819	0.9844	0.9844	0.9842	0.9846	0.9808	0.9705	0.9837	0.9844	0.9851
6_h	0.9869	0.9884	0.9885	0.9880	0.9886	0.9858	0.9754	0.9882	0.9886	0.9892
2_d	0.9758	0.9805	0.9810	0.9803	0.9805	0.9763	0.9583	0.9811	0.9815	0.9816
3_g	0.9805	0.9833	0.9834	0.9809	0.9835	0.9785	0.9637	0.9833	0.9839	0.9840
4_g	0.9798	0.9816	0.9815	0.9806	0.9815	0.9782	0.9649	0.9815	0.9821	0.9822
8_d	0.9734	0.9780	0.9780	0.9773	0.9781	0.9730	0.9600	0.9779	0.9782	0.9790
3_d	0.9772	0.9835	0.9831	0.9830	0.9834	0.9769	0.9593	0.9833	0.9833	0.9849

TABLE III
THE T-VALUE AND T-TABLE REFERENCE VALUE OF EACH U-NET VARIANT WHEN COMPARED WITH THE DEFAULT U-NET CONFIGURATION. EXPERIMENTS THAT YIELD SIGNIFICANTLY DIFFERENT PERFORMANCE THAN THE DEFAULT U-NET ARE HIGHLIGHTED IN BOLD FONT.

	residual	dense	dilate	side	**conv1**	**linear**	no skip	par	5 level
t-value	0.8332	0.9875	0.5603	1.0735	**2.3211**	**9.2258**	0.4956	1.3065	1.8436
t-table	1.9872	1.9876	1.9872	1.9876	**1.9876**	**1.9949**	1.9876	1.9879	1.9879

REFERENCES

[1] Budai, A., Bock, R., Maier, A., Hornegger, J., Michelson, G.: Robust Vessel Segmentation in Fundus Images. IJBI (2013)

[2] Kingma, D.P., Ba, J.: Adam: A Method for Stochastic Optimization. arXiv:1412.6980 (2014)

[3] Student: The probable error of a mean. Biometrika pp. 1–25 (1908)
